# Reappraisal of Real‐World Management of Acute Cholecystitis in Elderly Patients Based on the Adherence to Tokyo Guidelines 2018 (TG18): A Multicenter Study on Anzu HPB Surgical Meeting

**DOI:** 10.1002/ags3.70201

**Published:** 2026-02-19

**Authors:** Yuta Kobayashi, Shojiro Hata, Ryo Oikawa, Tetsuya Nakazato, Hiroki Sakata, Hirokazu Yamaguchi, Yutaka Suzuki, Yoshihiro Sakamoto

**Affiliations:** ^1^ Department of Gastroenterological Surgery Showa General Hospital Kodaira Japan; ^2^ Department of Surgery Tokyo Metropolitan Institute for Geriatrics and Gerontology Tokyo Japan; ^3^ Department of Surgery Kanto Rosai Hospital Kawasaki Japan; ^4^ Department of Gastroenterological Surgery Kyorin University Suginami Hospital Tokyo Japan; ^5^ Department of Hepato‐Biliary‐Pancreatic Surgery Kyorin University Hospital Tokyo Japan

**Keywords:** cholecystitis, elderly, surgery, TG18

## Abstract

**Background:**

Although the Tokyo Guidelines 2018 (TG18) recommend optimal management for acute cholecystitis (AC) using an age‐adjusted index, the implementation and outcomes of treatment strategies in elderly patients with AC remain unclear.

**Methods:**

A total of 461 patients aged ≥ 70 years with AC from five institutions were retrospectively analyzed, including those who underwent early surgery (*n* = 247) and later, elective surgery with (*n* = 114) or without (*n* = 100) preoperative gallbladder drainage. Patients were classified into three groups based on treatment strategy: A (consistent with TG18 recommendations, *n* = 206), B (more aggressive than TG18 recommendations, *n* = 135), and C (more conservative than TG18 recommendations, *n* = 120). Multivariate analyses were performed to identify potential surgical complication predictors.

**Results:**

Among Grade I/II AC patients, overall complication rates in Groups A, B, and C were 16.4%, 34.0%, and 8.3%, respectively (A vs. B: *p* < 0.001, A vs. C: *p* = 0.052) and major complication rates were 3.1%, 9.4%, and 5.0%, respectively (A vs. B: *p* = 0.029, A vs. C: *p* = 0.382). Laparoscopic approaches were most frequent in Group C (A, 59.7% vs. B, 22.6% vs. C, 96.6%, *p* < 0.001). Anticoagulant/antiplatelet therapy independently predicted major complications (odds ratio 3.10, *p* = 0.023).

**Conclusion:**

In the present trial, treatment for AC was consistent with TG18 recommendations in 45% of patients. Compared with TG18‐aligned treatment, more conservative treatment was associated with comparable morbidities, while more aggressive treatment was associated with a higher incidence of major morbidities.

## Graphical abstract

This multicenter retrospective study analyzes real‐world management of acute cholecystitis in patients aged ≥ 70 years across five institutions, focusing on adherence and intentional deviation from TG18 recommendations. By classifying treatment strategies as TG18‐accordant, more aggressive, or more conservative, we reappraise current practice patterns. More conservative strategies achieved postoperative outcomes comparable to TG18‐concordant treatment, whereas more aggressive surgical intervention tended to be associated with a higher incidence of major morbidity, and anticoagulant or antiplatelet therapy was identified as an independent risk factor for major complications.

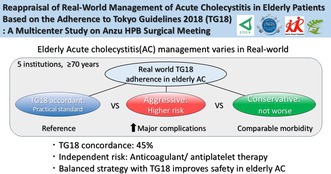

## 1 Introduction

The Tokyo Guidelines 2018 (TG18) for the management of acute cholecystitis (AC) have been widely recognized for their clinical utility and achieved global acceptance [1, 2, 3, 4]. In the TG18, a treatment algorithm utilizes an age‐adjusted index and the American Society of Anesthesiologists Physical Status (ASA‐PS) [5]. The severity of AC is classified into three categories, and treatment strategies for each category are proposed based on the age of the patient, number of comorbidities, and ASA‐PS score. Immediate cholecystectomy is recommended in relatively younger patients with mild to moderate TG18 severity gradings of AC I/II who have good performance status (ASA‐PS ≤ 2) and few comorbidities. By contrast, conservative disease management followed by cholecystectomy is recommended in elderly patients with a higher surgical risk, those with multiple comorbidities, or patients with a severe AC Grade III. Notably, because the age‐adjusted Charlson Comorbidity Index (CCI) increases with advancing age, the TG18 inherently tends to recommend more conservative treatment strategies as patients grow older. However, in real‐world practice, it is unclear whether treatments for AC in elderly patients are performed based on the recommendations in the TG18. Clinicians tend to deviate from guideline‐recommended strategies, being either overly conservative out of concern for age‐related risks or excessively invasive because of inadequate preoperative assessment.

The present study aimed to appraise the diversity of real‐world treatment strategies for AC in elderly patients, who often have several comorbidities and surgical risks, through a multicenter study in Tokyo.

## 2 Methods

### 2.1 Study Population

**FIGURE 1:**
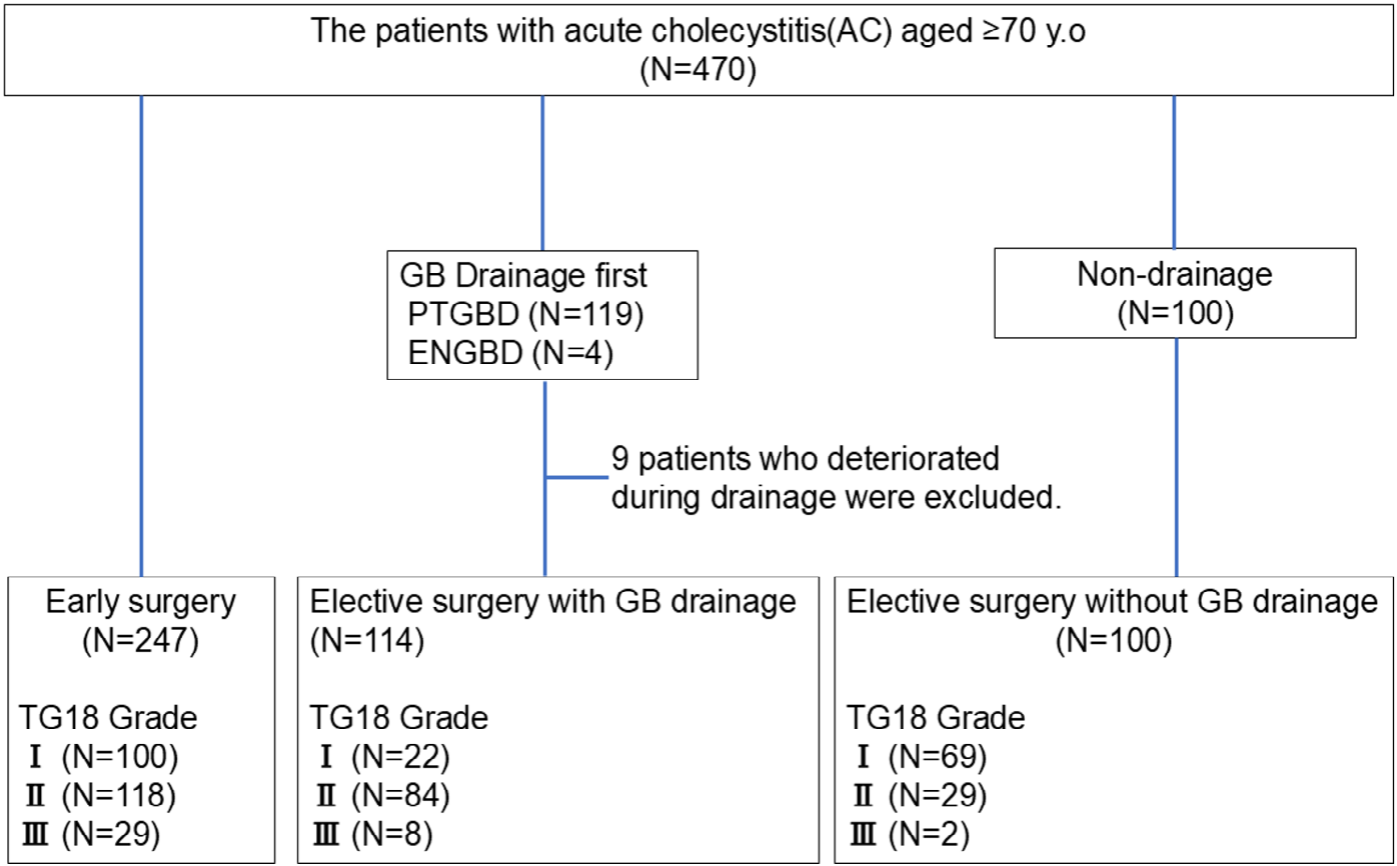
Flow diagram of patient selection.

The Anzu HPB Surgical Meeting Research Group consists of five institutions: Showa General Hospital, Kyorin University Hospital, Kyorin University Suginami Hospital, Tokyo Metropolitan Institute for Geriatrics and Gerontology, Kanto Rosai Hospital. Patients diagnosed with AC who underwent surgery between 2015 and 2024 were included in this study; 470 patients over 70 years old were identified for inclusion. Of these, nine patients who deteriorated during preoperative gall bladder (GB) drainage were excluded. Therefore, 461 patients were included in the study. Among them, 247 underwent early surgery within 48 h of admission [6, 7] 114 underwent elective surgery following preoperative GB drainage, and the remaining 100 underwent elective surgery without preoperative GB drainage (Figure 1).

This retrospective study utilized a newly updated database that was originally constructed for the previous study conducted by the same research group, with additional cases incorporated for the present analysis [8]. All analyses in the current study were performed in accordance with the ethical guidelines for clinical research in Japan and in compliance with the declaration of Helsinki, after obtaining approval from the Research Ethics Committee of Kyorin University, Tokyo, Japan (protocol #825).

### 2.2 Treatment Strategy and Surgical Indication

The treatment policies and strategies for AC at the five participating institutions have been reported previously, and reflect differences in institutional resources and clinical policies [8]. In principle, management was conducted in accordance with the TG18. Patients with a TG18 severity Grade of I or II who were deemed suitable for surgery under general anesthesia based on ASA‐PS, age, comorbidities, and overall condition underwent early surgery. For patients with a TG18 severity grade of III, stabilization of organ dysfunction through intensive care was initially attempted, and surgery was considered only if the patient was subsequently deemed surgically fit. Nevertheless, because of optimization of both human and institutional resources, conservative treatment with antibiotics was prioritized, or preoperative GB drainage procedures such as percutaneous transhepatic GB drainage or endoscopic retrograde GB drainage followed by elective surgery were employed instead [9]. The interval between drainage and subsequent surgery varied according to each institution's policy. The choice of surgical approach, whether open or laparoscopic, was also determined according to each institution's available resources. In all institutions, laparoscopic procedures were converted to open surgery when necessary, and bailout procedures were applied in cases where surgeons were struggling to obtain a critical view of safety (CVS) during surgery.

Bailout procedures were defined according to TG18 as subtotal cholecystectomy, performed irrespective of surgical approach, in cases where complete visualization of the CVS could not be achieved, with closure of the remnant gallbladder wall or cystic duct orifice.

### 2.3 The Conventional Surgical Indication Framework and Category of Treatment Concordance With the TG18

**FIGURE 2:**
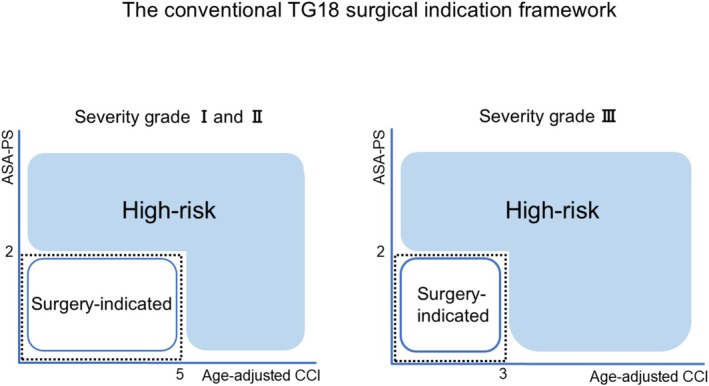
The conventional TG18 surgical indication framework.

Although the TG18 management algorithm for AC does not specifically address elderly patients, it defines surgical risk based on scores of CCI ≥ 6 or ASA‐PS ≥ 3 for patients with Grade I or II AC, and CCI ≥ 4 or ASA‐PS ≥ 3 for those with Grade III AC. In the context of treatment decision making for elderly patients, the conventional surgical indications for each severity grade can be broadly categorized, as illustrated in Figure 2, into a surgery‐indicated population (ASA‐PS ≤ 2 and CCI ≤ 5) and a high‐risk population (ASA‐PS ≥ 3 or CCI ≥ 6) [5]. Based on the real‐world diversity of AC management, we further categorized patients into three groups according to whether the treatment they received was in accordance with, or deviated from, TG18‐recommended surgical indications. Group A (treatment in accordance with TG18 recommendations) included patients who underwent early surgery in the surgery‐indicated population and patients who underwent elective surgery in the high‐risk population within the conventional TG18 surgical indication framework. Group B (more aggressive surgical intervention than TG18 recommendations) included patients in the high‐risk population who underwent early surgery. Group C (more conservative treatment than TG18 recommendations) included patients who met the conventional framework guidelines for early surgery, but who underwent delayed elective surgery instead.

### 2.4 Evaluation of Surgical Outcomes

The TG18 severity grade was determined based on physical findings, laboratory data, and imaging results at the time of symptomatic presentation. When there was no contraindication such as renal dysfunction, asthma, or allergies, contrast‐enhanced CT was generally performed for all cases. The status of gallstones was assessed on CT images, and the marked local inflammation findings included gangrenous cholecystitis, pericholecystic abscess, hepatic abscess, biliary peritonitis, and emphysematous cholecystitis. Postoperative outcomes were evaluated using the Clavien‐Dindo (C‐D) classification [10], with major complication defined as C‐D Grade 3 or higher and deaths within 90 days classified as Grade 5. Given the substantial selection bias inherent to retrospective analyses, sensitivity analyses were performed by restricting the primary analysis to patients with TG18 Grade I/II AC. Multivariate analysis was conducted to identify independent risk factors for postoperative complications. Patients with TG18 Grade III AC were excluded from this analysis, as treatment allocation in this subgroup was highly selective and likely to introduce significant selection bias.

### 2.5 Statistical Analysis

Continuous values are expressed as the median values (range) and compared using Wilcoxon's rank‐sum test. Categorical variables are expressed as numbers (%) and compared using Fisher's exact test or the chi‐squared test, as appropriate. *p* < 0.05 was considered as indicative of statistical significance. Additionally, to identify potential risk factors associated with postoperative complications, multivariate logistic regression with backward elimination was performed. To avoid overfitting, only factors that showed a statistically significant association with postoperative coarse at *p* < 0.1 were included in the final model. All the statistical analyses were conducted using the JMP software (version 14; SAS Institute Inc., Cary, NC) or IBM SPSS software (Ver. 26.0; SPSS Inc., IL, USA).

## 3 Results

### 3.1 Overview

**FIGURE 3:**
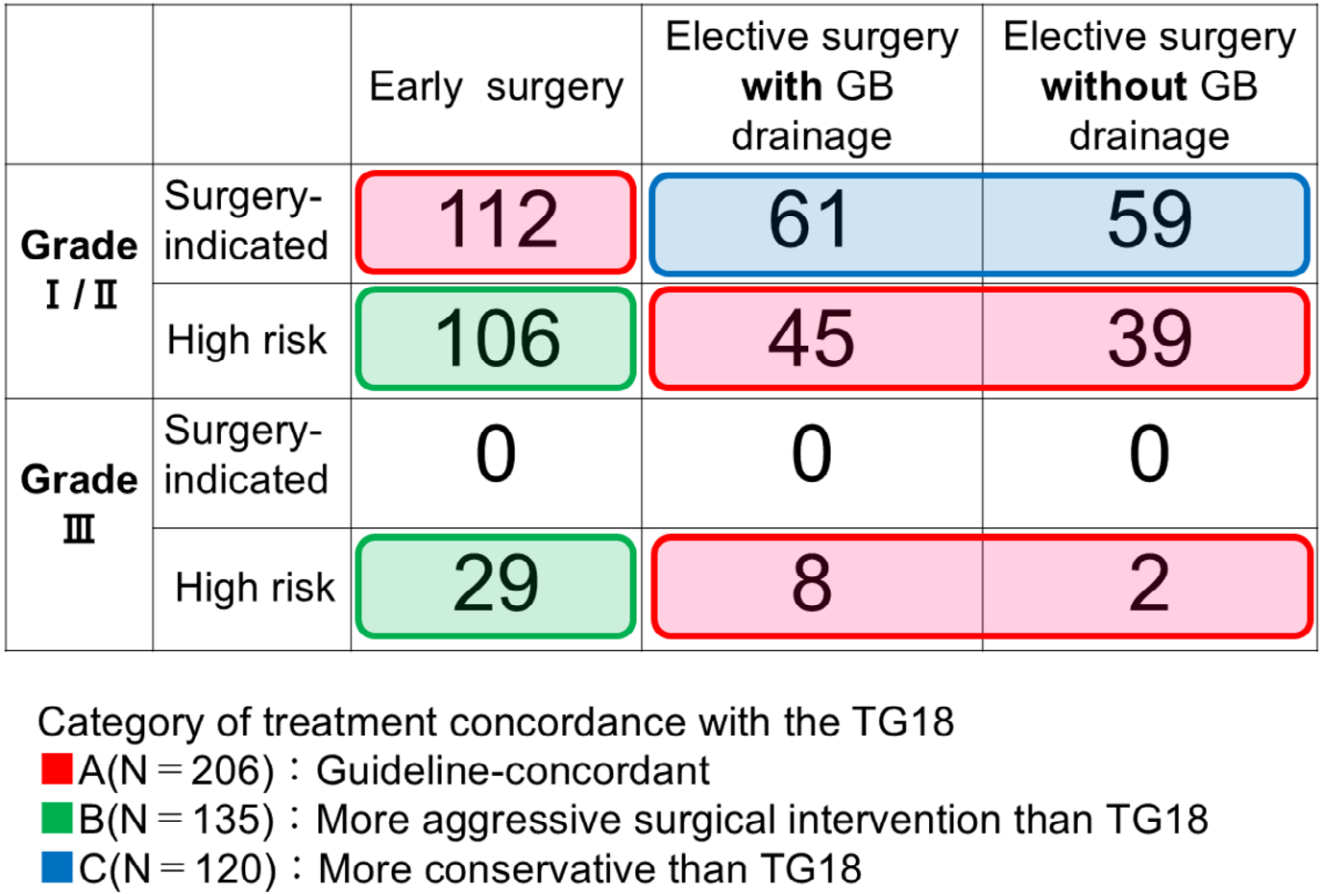
Distribution of treatment approach and categorization of treatment concordance.

The present analysis included 461 patients (290 men, 171 women; median age 78 years, range 70–95 years). Of the total cohort, 191 patients were classified as Grade I, 231 as Grade II, and 39 as Grade III according to the TG18 severity grading. From the perspective of treatment concordance with TG18 recommendations, 206 patients (44.7%) were classified into Group A, 135 patients (29.3%) into Group B, and 120 patients (26.0%) into Group C (Figure 3).

### 3.2 Patient's Characteristics and Postoperative Outcomes of Each Treatment

Patients with a TG18 severity grade of I or II were divided into a surgery‐indicated population (ASA‐PS ≤ 2 and CCI ≤ 5) and a high‐risk population (ASA‐PS ≥ 3 or CCI ≥ 6) based on the TG18 surgical indication framework; their characteristics and short‐term outcomes are summarized in Table 1. In both the surgery‐indicated and high‐risk populations, the proportion of Grade II AC was higher in the elective surgery with drainage subgroups, and Grade I AC was more frequent in the elective surgery without drainage subgroups. Laparoscopic surgery was more frequent in elective surgery groups, while conversion to open surgery was more common in early surgery groups for both surgery‐indicated and high‐risk populations. Bailout procedures occurred most often in drainage‐first elective surgery groups, and blood loss was significantly larger in the early surgery groups for both populations. Overall, postoperative complications were higher in early surgery groups than in elective surgery groups for both surgery‐indicated and high‐risk populations (19.6%, *p* = 0.027, and 34.0%, *p* = 0.002, respectively). By contrast, major complications did not differ significantly between early and elective surgery groups in the surgery‐indicated population (*p* = 0.657), and were nonsignificantly higher in the early surgery group than in the elective surgery groups in the high‐risk population (9.4% vs. 6.7% vs. 0%, *p* = 0.136).

**TABLE 1:** Patient's characteristics and short‐term outcomes of each surgical approach in severity Grade I/II.

	Surgery‐indicated population (PS ≤ 2 and CCI ≤ 5)				High‐risk population (PS ≥ 3 or CCI ≥ 6)			
	Early surgery	Elective surgery with GB drainage	Elective surgery without GB drainage	*p*	Early surgery	Elective surgery with GB drainage	Elective surgery without GB drainage	*p*
Category of treatment concordance	A	C	C		B	A	A	
Number of patients	112	61	59		106	45	39	
Age (years)	76 (70–93)	75 (70–92)	75 (70–88)	0.594	82 (70–93)	81 (70–90)	79 (70–89)	0.012
Gender (male/female)	67/45	38/23	38/21	0.836	69/37	27/18	25/14	0.836
BMI	23.2 (17.1–38.7)	23.4 (15.9–34.1)	24.1 (16.4–31.0)	0.572	22.0 (17.9–33.2)	23.3 (17.0–38.8)	22.7 (17.5–30.6)	0.034
ASA‐PS 1/2/3/4	16/96/0/0	11/50/0/0	3/56/0/0	0.090	0/53/52/1	4/26/15/0	0/14/25/0	0.003
Age‐adjusted CCI	5 (3–5)	5 (3–7)	4 (3–5)	0.161	6 (3–11)	6 (4–9)	6 (4–8)	0.192
Comorbidities and past history								
Hyper tension	49 (43.8)	34 (55.7)	38 (64.4)	0.030	69 (65.1)	28 (62.2)	29 (74.4)	0.464
Ischemic heart disease	5 (4.5)	3 (4.9)	2 (3.4)	0.913	19 (17.9)	8 (17.8)	11 (28.2)	0.356
Diabetes	15 (13.4)	13 (21.3)	9 (15.3)	0.392	36 (34.0)	24 (53.3)	10 (25.6)	0.021
Chronic respiratory disease	3 (2.7)	7 (11.5)	3 (5.1)	0.055	8 (7.6)	7 (15.6)	9 (23.1)	0.035
Use of anticoagulant/platelet drug	21 (18.8)	19 (31.2)	14 (23.7)	0.182	48 (45.3)	26 (57.8)	20 (51.3)	0.361
Use of steroid	1 (0.9)	1 (1.6)	1 (1.7)	0.873	5 (4.7)	3 (6.7)	3 (7.7)	0.761
History of abdominal surgery	33 (29.5)	21 (34.4)	18 (30.5)	0.793	37 (34.9)	18 (40.0)	9 (23.1)	0.242
Marked local inflammation^a^	24 (21.4)	13 (21.3)	3 (5.1)	0.017	30 (28.3)	10 (22.2)	2 (5.1)	0.012
Stone impaction of the GB neck	47 (42.0)	13 (21.7)	9 (15.3)	< 0.001	36 (34.0)	17 (37.8)	9 (23.1)	0.325
Stone fullness	3 (2.7)	5 (8.3)	7 (11.9)	0.059	4 (3.8)	1 (2.2)	2 (5.1)	0.778
Cystic duct stones	12 (10.8)	4 (6.7)	3 (5.1)	0.379	13 (12.3)	3 (6.7)	5 (12.8)	0.559
Preoperative ERCP	9 (8.0)	16 (26.2)	19 (32.2)	< 0.001	10 (9.4)	12 (26.7)	13 (33.3)	0.001
Preoperative EST	8 (7.1)	15 (24.6)	17 (28.8)	< 0.001	9 (8.5)	9 (20.0)	10 (25.6)	0.019
TG 18 severity Grade I/II^b^	46 (41.1)/66 (58.9)	12 (19.7)/49 (80.3)	43 (72.9)/16 (27.1)	< 0.001	54 (50.9)/52 (49.1)	10 (22.2)/35 (77.8)	26 (66.7)/13 (33.3)	< 0.001
Final operative approach								
Laparoscopic (completed)	45 (40.2)	59 (96.7)	57 (96.6)	< 0.001	82 (77.4)/24 (22.6)	7 (15.6)/38 (84.4)	5 (12.88)/34 (87.2)	< 0.001
Planned open	57 (50.9)	1 (1.7)	0	< 0.001	73 (68.9)	0	0	< 0.001
Converted to open surgery (%)	10 (18.2)	1 (1.7)	2 (3.4)	0.001	9 (27.3)	7 (15.6)	5 (12.8)	0.244
Bailout procedure	11 (9.8)	20 (32.8)	4 (6.8)	< 0.001	10 (9.4)	14 (31.1)	7 (18.0)	0.004
Injury of bile duct	0	1 (1.6)	0	0.245	1 (0.9)	1 (2.2)	0	0.601
Operative time, min	132 (23–316)	147 (54–234)	134 (42–283)	0.295	121 (56–223)	162 (76–309)	148 (46–285)	< 0.001
Blood lost, mL	125 (0–2490)	20 (0–511)	5 (0–2057)	< 0.001	150 (0–2078)	41 (0–831)	3 (0–1643)	< 0.001
Overall complication	22 (19.6)	7 (11.7)	2 (4.1)	0.027	36 (34.0)	6 (13.6)	2 (7.4)	0.002
Bile leakage	4 (3.6)	1 (1.6)	0	0.295	6 (5.7)	1 (2.2)	0	0.231
Abdominal abscess	2 (1.8)	2 (3.3)	2 (3.4)	0.759	6 (5.7)	2 (4.4)	0	0.321
Postoperative bleeding	1 (0.9)	1 (1.6)	0	0.623	1 (0.9)	2 (4.4)	1 (2.6)	0.381
Superficial SSI	7 (6.3)	0	2 (3.4)	0.123	9 (8.5)	2 (4.4)	1 (2.6)	0.360
Major complication^c^	3 (2.7)	3 (4.9)	3 (5.1)	0.657	10 (9.4)	3 (6.7)	0	0.136
Mortality	0	0	0		1 (0.9)^d^	0	0	0.671
Postoperative stay, days	7 (3–23)	5 (3–58)	4 (3–24)	< 0.001	8 (3–56)	6 (3–107)	5 (3–53)	< 0.001

Abbreviations: ASA‐PS, American association of anesthesia‐physical status; BMI, body mass index; CCI, Charlson comorbidity index; ERCP, Endoscopic retrograde cholangiopancreatography; EST, Endoscopic sphincterotomy; GB, gallbladder; SSI, Surgical site infection.

^a^ Image findings of gangrenous cholecystitis, pericholecystic abscess, hepatic abscess, biliary peritonitis and emphysematous cholecystitis.

^b^ Yokoe M et al. *J Hepatobiliary Pancreat Sci* 2017;24:338–345.

^c^ Clavien‐Dindo Grade III or greater.

^d^ One patient died from pancreatitis after ERCP.

In patients with a TG18 severity grade of III, laparoscopic surgery was significantly less frequent in the early surgery group than in the elective surgery group. Operative time and blood loss were significantly lower in the elective surgery group without preoperative drainage than in the elective surgery group with preoperative drainage. Major complications were predominantly observed in the early surgery group (34.5%), which included three cases of mortality. One patient died from colonic perforation associated with nonocclusive mesenteric ischemia, and two patients died from progressive disseminated intravascular coagulation and multiple organ failure secondary to systemic infection (Table S1).

Table 2 presents the patient characteristics and short‐term outcomes in each group according to treatment concordance with TG18 recommendations in patients with a TG18 severity grade of I or II. In the comparison between Group A and Group B, Group B had a higher proportion of patients with ASA‐PS scores of 3 or 4 and higher CCI scores (both *p* < 0.001). Laparoscopic surgery was more frequently performed in Group A than in Group B (59.7% vs. 22.6%, *p* < 0.001). By contrast, the overall complication rate (34.0% vs. 16.4%, *p* < 0.001) and major complication rate (9.4% vs. 3.1%, *p* = 0.029) were significantly higher in Group B than in Group A.

**TABLE 2:** Patient's characteristics and short‐term outcomes by category of treatment concordance with TG18 in Grade I/II.

	Group A: Accordant with TG18	Group B: More aggressive surgical intervention than TG18	Group C: More conservative than TG18	A vs. B vs. C, *p*	A vs. B, *p*	A vs. C, *p*
Number of patients	196	106	120			
Age (years)	78 (70–93)	82 (70–93)	75 (70–92)	< 0.001	< 0.001	0.006
Gender (male/female)	119/77	69/37	76/44	0.739	0.454	0.721
BMI	23.1 (17.0–38.8)	22.0 (17.9–33.2)	23.9 (15.9–34.1)	0.003	0.006	0.347
ASA‐PS 1/2/3/4	20 (10.2)/136 (69.4)/40 (20.4)/0	0/53 (50.0)/52 (49.1)/1 (0.9)	14 (11.7)/106 (88.3)/0/0	< 0.001	< 0.001	< 0.001
Age‐adjusted CCI	5 (3–9)	6 (3–11)	5 (3–7)	< 0.001	< 0.001	< 0.001
Comorbidities and past history						
Hyper tension	106 (54.1)	69 (65.1)	72 (60.0)	0.166	0.068	0.350
Ischemic heart disease	24 (12.2)	19 (17.9)	5 (1.2)	0.004	0.227	0.016
Diabetes	49 (25.0)	36 (34.0)	22 (18.3)	0.026	0.109	0.211
Chronic respiratory disease	19 (9.7)	8 (7.6)	10 (8.3)	0.804	0.674	0.841
Use of anticoagulant/platelet drug	67 (34.2)	48 (45.3)	33 (27.5)	0.019	0.063	0.262
Use of steroid	7 (3.6)	5 (4.7)	2 (1.7)	0.426	0.759	0.491
History of abdominal surgery	60 (30.6)	37 (34.9)	39 (32.5)	0.746	0.519	0.803
Marked local inflammation^a^	36 (18.4)	30 (28.3)	16 (13.3)	0.016	0.058	0.276
Stone impaction of the GB neck	73 (37.2)	36 (34.0)	22 (18.5)	0.002	0.617	< 0.001
Stone fullness	6 (3.1)	4 (3.8)	12 (10.1)	0.020	0.746	0.013
Cystic duct stones	20 (10.3)	13 (12.3)	7 (5.9)	0.237	0.700	0.216
Preoperative ERCP	34 (17.4)	10 (9.4)	35 (29.2)	< 0.001	0.087	0.017
Preoperative EST	27 (13.8)	9 (8.5)	32 (26.7)	< 0.001	0.197	0.007
TG 18 severity grade I/II/^b^	82 (41.8)/114 (58.2)	54 (50.9)/52 (49.1)	55 (45.8)/65 (54.2)	0.313	0.146	0.559
Final operative approach						
Laparoscopic (completed)	117 (59.7)	24 (22.6)	116 (96.7)	< 0.001	< 0.001	< 0.001
Planned open	57 (29.1)	73 (68.9)	1 (0.8)	< 0.001	< 0.001	< 0.001
Converted to open surgery (%)	22 (15.8)	9 (27.3)	3 (2.5)	< 0.001	0.135	< 0.001
Bailout procedure	32 (16.3)	10 (9.4)	24 (20.0)	0.087	0.118	0.449
Injury of bile duct	1 (0.5)	1 (0.9)	1 (0.7)	0.897	1.000	1.000
Operative time, min	140 (23–316)	121 (56–223)	139 (42–283)	0.014	0.005	0.705
Blood lost, mL	57 (0–2490)	150 (0–2078)	10 (0–2057)	< 0.001	< 0.001	< 0.001
Overall complication	30 (16.4)	36 (34.0)	9 (8.3)	< 0.001	< 0.001	0.052
Bile leakage	5 (2.6)	6 (5.7)	1 (0.8)	0.088	0.203	0.414
Abdominal abscess	4 (2.0)	6 (5.7)	4 (3.3)	0.245	0.105	0.484
Postoperative bleeding	4 (2.0)	1 (0.9)	1 (0.8)	0.605	0.660	0.653
Superficial SSI	10 (5.1)	9 (8.5)	2 (1.7)	0.062	0.320	0.142
Major complication^c^	6 (3.1)	10 (9.4)	6 (5.0)	0.059	0.029	0.382
Mortality	0	1 (0.9)	0	0.226	0.351	
Postoperative stay, days	6 (3–107)	8 (3–56)	5 (3–58)	< 0.001	< 0.001	< 0.001

Abbreviations: ASA‐PS, American association of anesthesia‐physical status; BMI, body mass index; CCI, Charlson comorbidity index; ERCP, Endoscopic retrograde cholangiopancreatography; EST, Endoscopic sphincterotomy; GB, gallbladder; SSI, Surgical site infection.

^a^ Image findings of gangrenous cholecystitis, pericholecystic abscess, hepatic abscess, biliary peritonitis and emphysematous cholecystitis.

^b^ Yokoe M et al. *J Hepatobiliary Pancreat Sci* 2017;24:338–345.

^c^ Clavien‐Dindo Grade III or greater.

In the comparison between Group A and Group C, patients in Group C were significantly younger and tended to have lower ASA‐PS and CCI scores. In addition, the laparoscopic approach was more frequently performed in Group C than in Group A (96.6% vs. 60.2%, *p* < 0.001), with a significantly lower conversion rate to open surgery. Overall complications were less frequent in Group C than in Group A (8.3% vs. 17.3%, *p* = 0.037), whereas there were no significant differences in major complications or length of hospital stay. One patient in Grade B died from pancreatitis after endoscopic retrograde cholangiopancreatography.

### 3.3 Predictive Factor of Postoperative Complications

Table 3 summarizes the potential risk factors for postoperative complications among the variables associated with perioperative conditions in patients with a TG18 severity grade of I or II. The use of anticoagulant/antiplatelet drugs was identified as an independent predictor of major complications (odds ratio 3.10, *p* = 0.023), while age‐adjusted CCI and open surgery were independent predictors of overall complications. Univariate analyses were performed to assess the association between the use of anticoagulant/antiplatelet drugs and the risk of postoperative complications, including bile leakage, intra‐abdominal abscess development, postoperative bleeding, and surgical site infection. Among these, the strongest association was with bile leakage (odds ratio 4.38, *p* = 0.015, 95% confidence interval 1.32–14.5). Bile leakage occurred significantly more frequently in patients with anticoagulant/antiplatelet drug use (nine of 161 patients, 5.6%) than in those without (four of 300 patients, 1.3%; *p* = 0.015). In a multivariable analysis limited to patients with Grade I/II AC in Groups A and B, conducted to explore confounding by surgical difficulty and treatment selection, Group B (odds ratio 5.21, *p* = 0.016) and increased intraoperative blood loss (odds ratio 1.01, *p* = 0.022) were independently associated with a higher incidence of major postoperative complications (Table S3).

**TABLE 3:** Multivariate analysis to identify the perioperative risk for severity Grade I and II.

	*p* ^a^	OR	95% CI
Major complication			
Use of anticoagulant/platelet drug	0.023	3.10	1.17–8.23
Overall complication			
Age adjusted CCI	0.001	1.47	1.18–1.84
Open surgery	0.024	1.85	1.09–3.13

*Note:* Multivariate logistic regression was applied with stepwise backward selection. Initially, all factors presenting with *p* < 0.5 in the univariate analysis were included in the model. Then factors that showed no or limited statistically significant association (*p* > 0.1) with overall complication, major complication and nonhome discharge adjusted for the remaining factors in the model were deleted from the model in stepwise fashion. The 18 tested variables for overall complication and major complication were as follows: age, sex(male), BMI, ASA‐PS, age‐adjusted CCI, diabetes, chronic respiratory disease, ischemic heart disease, use of anticoagulant/platelet drug, Use of steroid, History of abdominal surgery, Marked local inflammation, Stone impaction of the GB neck, Preoperative EST, TG 18 severity Grade I or II, surgical procedure (open or laparoscopic), preoperative GB drainage, emergency or elective surgery and institutions.

Abbreviations: 95% CI, 95% confidence interval; ASA‐PS, American association of anesthesia‐physical status; BMI, body mass index; CCI, Charlson comorbidity index; GB, gallbladder; TG18, Tokyo guideline 18.

^a^ Based on likelihood test adjusted for the other factors in the final model.

### 3.4 Postoperative Outcomes of the Patients Undergoing Elective Surgery Stratified by the Use of Anticoagulant/Platelet Therapy

Patients who underwent elective surgery after initial GB drainage and those who proceeded directly to elective surgery without drainage were stratified and compared according to the use of anticoagulant/antiplatelet therapy (Table S2). Both groups achieved laparoscopic completion rates exceeding 90%. The overall complication rates were similar, as were the rates of major complications. Postoperative hospital stay was slightly longer in patients receiving anticoagulant/antiplatelet therapy.

## 4 Discussion

The present study investigated real‐world treatments for AC in elderly patients and compared them with recommendations from the TG18 in five suburban Tokyo hospitals. Only 45% of patients with AC were treated in accordance with TG18 recommendations, while 29% of patients underwent more aggressive surgical intervention than that recommended in the TG18, which tended to be associated with increased major morbidities. By contrast, 26% of patients underwent more conservative treatment than that recommended in the TG18, specifically disease management followed by elective, minimally invasive cholecystectomy, which tended to be associated with an acceptable level of morbidities. This variety of strategies may reflect the difficulty of implementing the treatments for AC recommended in the TG18, and real‐world strategies were likely determined by the clinical policy and number of staff in each hospital.

Although the TG18 algorithm has been extensively validated by many researchers since its release, treatment recommendations for elderly patients with multiple comorbidities have not been clearly established. Using ASA‐PS and age‐adjusted CCI scores to refine surgical indications has effectively served as a safeguard against overly aggressive surgery in elderly or medically fragile patients and has helped avoid unnecessary procedural risk. However, in real‐world clinical practice, management patterns vary across institutions because of the consideration of age‐related risks [11, 12, 13], even when guideline‐recommended early surgery would otherwise be appropriate. By contrast, inadequate preoperative assessment in certain cases may lead to unexpectedly aggressive treatment despite substantial comorbidities [14, 15, 16]. Therefore, the same therapeutic approach could be considered appropriate, overly aggressive, or overly conservative.

Several prior studies have attempted to validate the TG18, including in elderly populations and in the context of treatment selection. Although higher age and compromised physical or nutritional status are generally considered surgical risk factors [17, 18], the other has reported acceptable outcomes with TG18‐based management even among super‐elderly patients [19]. However, few have examined deviations from TG18‐recommended strategies and evaluated such deviations in relation to actual clinical decision making and outcomes. Our findings revealed a distinct discrepancy between conventional TG18 treatment strategies and real‐world clinical practice: only 45% of patients received treatment concordant with TG18 recommendations (Group A), whereas the remaining cases were almost evenly divided between the more aggressive surgical intervention group (Group B) and the more conservative treatment group (Group C). Group B patients were characterized by more severe local inflammatory imaging findings and a higher proportion of Grade III AC cases. The striking imaging findings, coupled with the AC severity grade, may obscure a proper assessment of underlying comorbidities and potentially lead to overly aggressive surgical decisions. Another reason for favoring one‐stage surgery in elderly patients may be the practical difficulty they often face in managing a percutaneous transhepatic GB drainage tube at home during the waiting period. By contrast, patients in Group C generally exhibited lower ASA‐PS and CCI scores and had the highest proportion of Grade I AC cases. These patients were, in principle, suitable candidates for early surgery according to the TG18. The choice of delayed surgery was likely driven by institutional capacity for emergency operations and limitations in available resources. Although these patients had the highest laparoscopic completion rate and complication rates comparable to those of Group A, the extended waiting time and associated costs cannot be overlooked.

Particular caution is warranted when determining the suitability of early surgery for patients receiving anticoagulant therapy. Even when high‐risk patients on anticoagulant/antiplatelet therapy were managed conservatively and underwent delayed elective surgery, their postoperative outcomes were not different from those without anticoagulant/antiplatelet therapy. In this cohort, anticoagulant/antiplatelet drug use was significantly associated with increased odds of postoperative bile leakage. Generally, bailout procedures are thought to increase the risk of bile leakage because they often preclude complete cystic duct closure [20, 21, 22]. Although patients on anticoagulant/antiplatelet drugs were more likely to undergo bailout procedures, the difference was not statistically significant in this study (8/69, 11.6% vs. 13/149, 8.7%; *p* = 0.622).

Among Grade III AC patients, even after stabilization of their systemic condition through intensive care, those undergoing early surgery had a substantially higher incidence of major complications, including three deaths. The patients who underwent early surgery were those who responded favorably to initial intensive care, whereas those in the elective surgery group consisted of patients who survived the acute phase. Consequently, an extremely strong selection bias is inherent in comparisons between these groups. Although favorable postoperative outcomes of elective surgery in Grade III, surgical indication in Grade III patients must always be considered in comparison with alternative conservative treatment strategies and require the most careful preoperative and perioperative assessment.

**FIGURE 4:**
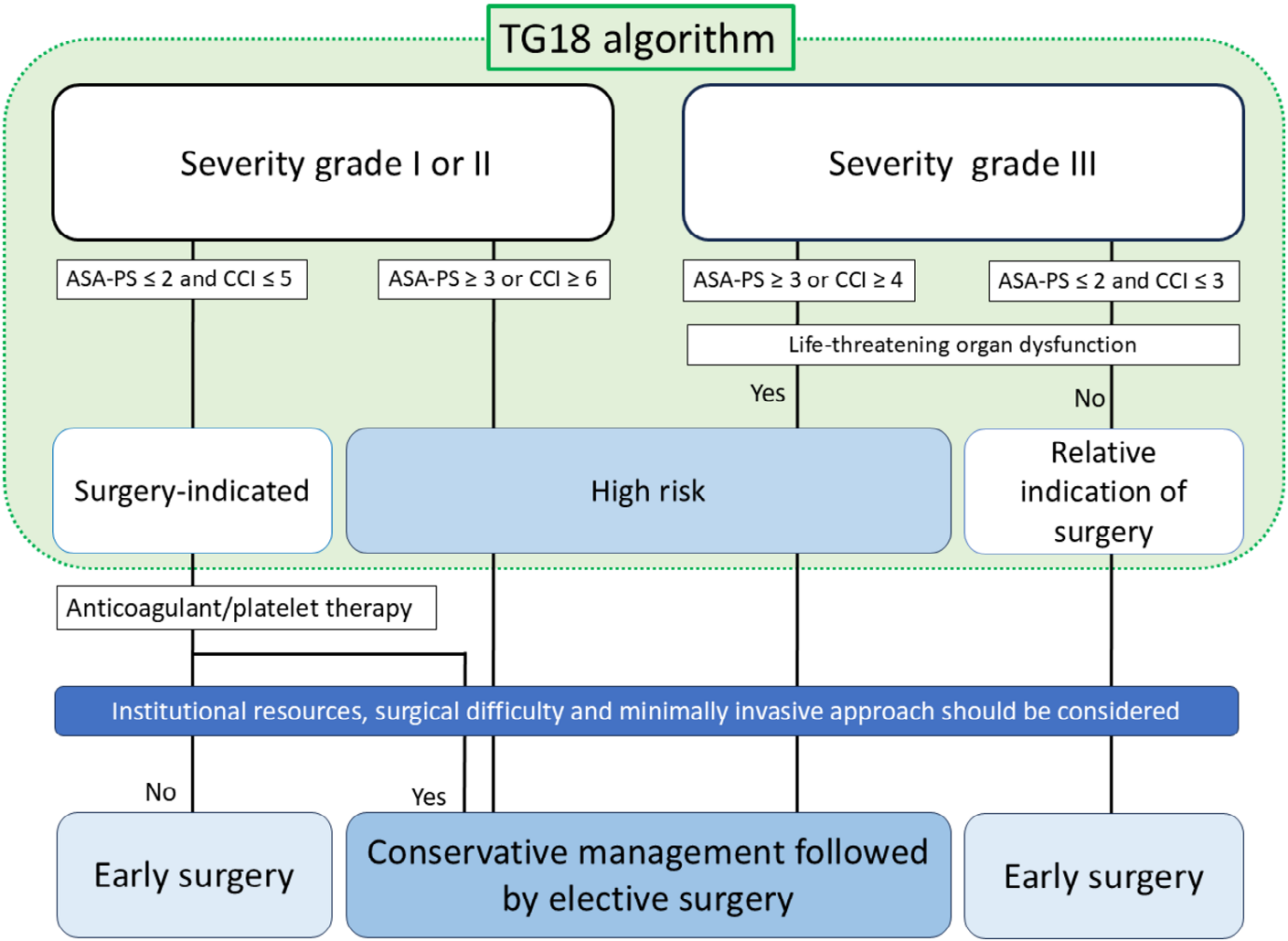
Proposed clinical flowchart for the management of acute cholecystitis in elderly patients.

Given the substantial variability in real‐world treatment selection, it is important to adhere to the TG18 algorithm while also considering anticipated surgical difficulty, the feasibility and optimal timing of a minimally invasive approach, and the institutional resources available at the time of decision‐making. Based on these considerations, we propose a modified TG18‐based clinical flowchart to support practical decision‐making in elderly patients with AC (Figure 4).

This study has several limitations. First, its retrospective nature inevitably introduces selection and information bias. In particular, the classification of treatment strategy groups (A, B and C) may have been influenced by clinical context and institutional policies and practice patterns that could not be fully captured in a retrospective design. However, this heterogeneity also serves as a strength because it reflects real‐world variations in clinical practice. Second, postoperative mid‐ to long‐term biliary complications, as well as biliary events occurring during the waiting period in Group C, were not evaluated in this study and warrant further investigation. In addition, a high proportion of emergency cholecystectomies were performed as open procedures because of resource limitations, indicating that many patients did not benefit from minimally invasive strategies [23]. Nevertheless, given the current healthcare structure in Japan, it is not feasible to centralize the management of such a common disease as AC. Each institution must therefore make the best use of its available resources to optimize patient care within its context [24].

In conclusion, our overview of real‐world management of elderly patients with AC revealed frequent deviations from TG18 recommendations. Our results suggest that TG18 treatment strategies are broadly safe and applicable to elderly patients with AC.

## Author Contributions

**Yuta Kobayashi:** conceptualization, methodology, data curation, investigation, validation, visualization, writing – original draft, writing – review and editing. **Shojiro Hata:** data curation. **Ryo Oikawa:** data curation. **Tetsuya Nakazato:** data curation. **Hiroki Sakata:** data curation. **Hirokazu Yamaguchi:** data curation. **Yutaka Suzuki:** data curation, validation, supervision. **Yoshihiro Sakamoto:** validation, supervision.

## Funding

The authors have nothing to report.

## Ethics Statement

Approved by the Research Ethics Committee of Kyorin University (protocol #825).

## Consent

IC was waived due to the retrospective nature of the study.

## Conflicts of Interest

The authors declare no conflicts of interest.

## Supporting information


**Table S1:** Patient's characteristics and short‐term outcomes of each surgical approach in severity Grade III.

**Table S2:** Postoperative outcomes of the patients undergoing elective surgery stratified by anticoagulant/platelet therapy.

**Table S3:** Multivariate analysis to identify the risk in Group A and B for severity Grade I and II.
