# Supplementary material for: Reappraisal of Real‐World Management of Acute Cholecystitis in Elderly Patients Based on the Adherence to Tokyo Guidelines 2018 (TG18): A Multicenter Study on Anzu HPB Surgical Meeting
Source: Ann Gastroenterol Surg. 2026 Feb 19;10(4):1250–60. doi: 10.1002/ags3.70201 (PMC13327017; doi:10.1002/ags3.70201)
Supplement: Supplementary file 1 — Table S1: Patient's characteristics and short‐term outcomes of each surgical approach in severity Grade III. [file AGS3-10-1250-s002.docx]

| **Supplementary Table1. Patient’s characteristics and short-term outcomes of each surgical approach in severity grade Ⅲ** | | | | |
| --- | --- | --- | --- | --- |
|  | High-risk population (PS≥3 or CCI≥6) | | | |
|  | Early surgery | Elective surgery  with GB drainage | Elective surgery without GB drainage | p |
| Category of treatment concordance | B | A | A |  |
| Number of patients | 29 | 8 | 2 |  |
| Age (years) | 82（71-95） | 78（70-93） | 75（72-78） | 0.146 |
| Gender (Male/Female) | 17/12 | 7/1 | 2/0 | 0.182 |
| BMI | 22.2(17.5-25.8) | 23.8(21.6-32.2) | 20.3(20.0-20.6) | 0.087 |
| ASA-PS 1/ 2/ 3/ 4 | 0/10(34.5)/16(55.2)/3(10.3) | 0/4(50.0)/4(50.0)/0 | 0/2(100)/0/0 | 0.367 |
| Age-adjusted CCI | 6(4-10) | 5(4-6) | 5 | 0.006 |
| Comorbidities and past history |  |  |  |  |
| Hyper tension | 18(62.1) | 6(75.0) | 1(50.0) | 0.727 |
| Ischemic heart disease | 4(13.8) | 0 | 0 | 0.464 |
| Diabetes | 5(17.2) | 2(25.0) | 1(50.0) | 0.508 |
| Chronic respiratory disease | 4(13.8) | 0 | 0 | 0.464 |
| Use of anticoagulant/ platelet drug | 12(41.4) | 1(12.5) | 0 | 0.182 |
| Use of steroid | 1(3.5) | 0 | 0 | 0.838 |
| History of abdominal surgery | 8(27.6) | 1(12.5) | 1(50.0) | 0.500 |
|  |  |  |  |  |
| Marked local inflammation ^a^ | 11(37.9) | 4(50.0) | 1(50.0) | 0.799 |
| Stone impaction of the GB neck | 7(24.1) | 4(50.0) | 0 | 0.235 |
| Stone fullness | 0 | 0 | 0 |  |
| Cystic duct stones | 1(3.5) | 1(12.5) | 0 | 0.557 |
| Preoperative ERCP | 1(3.5) | 0 | 1(50.0) | 0.012 |
| Preoperative EST | 1 | 0 | 0 | 0.838 |
|  |  |  |  |  |
| Final operative approach |  |  |  |  |
| Laparoscopic (completed) | 3(10.3) | 5(62.5) | 2(100) | <0.001 |
| Planned open | 25(86.2) | 0 | 0 | <0.001 |
| Converted to open surgery (%) | 1(25.0) | 3(37.5) | 0 | 0.566 |
| Bailout procedure | 7(24.1) | 3(37.5) | 0 | 0.518 |
| Injury of bile duct | 0 | 0 | 0 |  |
| Operative time, min | 130(82-255) | 177(53-244) | 83(74-91) | 0.039 |
| Blood lost, ml | 250(0-3225) | 48(0-490) | 7(3-10) | 0.021 |
| Overall complication | 15(51.7) | 3(42.9) | 0 | 0.562 |
| Bile leakage | 1(3.5) | 0 | 0 | 0.838 |
| Abdominal abscess | 3(10.3) | 0 | 0 | 0.571 |
| Postoperative bleeding | 0 | 0 | 0 |  |
| Superficial SSI | 1(3.5) | 1(12.5) | 0 | 0.557 |
| Major complication ^b^ | 10(34.5) | 0 | 0 | 0.098 |
| Mortality | 3(10.3)^c^ | 0 | 0 | 0.571 |
| Postoperative stay, days | 12(4-64) | 7(3-35) | 5(4-5) | 0.010 |
|  |  |  |  |  |
| Abbreviation: BMI, body mass index; ASA-PS, American association of anesthesia-physical status;  CCI, Charlson comorbidity index; GB, gallbladder; ERCP, Endoscopic retrograde cholangiopancreatography; EST, Endoscopic sphincterotomy；SSI, Surgical site infection  a: Image findings of gangrenous cholecystitis, pericholecystic abscess, hepatic abscess, biliary peritonitis and emphysematous cholecystitis.  b: Clavien-Dindo grade III or greater.  c: One patient died from colonic perforation associated with nonocclusive mesenteric ischemia (NOMI), and two patients died from progression of disseminated intravascular coagulation (DIC) and multiple organ failure caused by systemic infection; all three were classified as TG18 severity Grade 3. | | | |  |
