# Supplementary material for: Reappraisal of Real‐World Management of Acute Cholecystitis in Elderly Patients Based on the Adherence to Tokyo Guidelines 2018 (TG18): A Multicenter Study on Anzu HPB Surgical Meeting
Source: Ann Gastroenterol Surg. 2026 Feb 19;10(4):1250–60. doi: 10.1002/ags3.70201 (PMC13327017; doi:10.1002/ags3.70201)
Supplement: Supplementary file 2 — Table S2: Postoperative outcomes of the patients undergoing elective surgery stratified by anticoagulant/platelet therapy. [file AGS3-10-1250-s001.docx]

| **Supplementary Table.2. Postoperative outcomes of the patients undergoing elective surgery stratified by anticoagulant/ platelet therapy** | | | |
| --- | --- | --- | --- |
|  | Elective surgery with /without GB drainage | | |
|  | Absence of anticoagulant/ platelet therapy | With anticoagulant/  platelet therapy | P value |
| Number of patients | 134 | 80 |  |
| TG 18 severity grade Ⅰ/ Ⅱ/ Ⅲ ^a^ | 64(47.8)/61(45.5)/9(6.7) | 27(33.8)/52(65.0)/1(1.3) | 0.011 |
| Preoperative GB drainage | 68(50.8) | 46(57.5) | 0.396 |
| Final operative approach |  |  |  |
| Laparoscopic (completed) | 122(91.7) | 72(90.0) | 0.805 |
| Planned open | 1(0.75) | 0 | 0.805 |
| Conversion to open surgery (%) | 10(7.6) | 8(10.0) | 0.528 |
| Bailout procedure | 25(18.7) | 23(28.8) | 0.093 |
| Intraoperative injury of bile duct | 1(0.8) | 1(1.3) | 1.000 |
| Operative time, min | 147(46-283) | 142(42-309) | 0.292 |
| Blood lost, ml | 8(0-2057) | 14(0-1643) | 0.235 |
| Overall complication | 12(10.3) | 8(11.1) | 1.000 |
| Bile leakage | 0 | 2(2.5) | 0.139 |
| Abdominal abscess | 6(4.5) | 0 | 0.086 |
| Postoperative bleeding | 2(1.5) | 2(2.5) | 0.631 |
| Superficial SSI | 5(3.7) | 1(1.3) | 0.414 |
| Major complication ^b^ | 5(3.7) | 4(5.0) | 0.731 |
| Mortality | 0 | 0 |  |
| Postoperative stay, days | 4(3-107) | 6(3-58) | <0.001 |
| a: Yokoe M et al. J Hepatobiliary Pancreat Sci 2017;24:338-345.  b: Clavien-Dindo grade III or greater. | | | |
