# Supplementary material for: Reappraisal of Real‐World Management of Acute Cholecystitis in Elderly Patients Based on the Adherence to Tokyo Guidelines 2018 (TG18): A Multicenter Study on Anzu HPB Surgical Meeting
Source: Ann Gastroenterol Surg. 2026 Feb 19;10(4):1250–60. doi: 10.1002/ags3.70201 (PMC13327017; doi:10.1002/ags3.70201)
Supplement: Supplementary file 3 — Table S3: Multivariate analysis to identify the risk in Group A and B for severity Grade I and II. [file AGS3-10-1250-s003.docx]

| **Supplementary Table3. Multivariate analysis to identify the risk in Group A and B**  **for severity grade Ⅰ and Ⅱ** | | | |
| --- | --- | --- | --- |
|  | p value† | OR | 95%CI |
| Major complication | | | |
| Group B | 0.016 | 5.21 | 1.36-20.0 |
| Blood lost | 0.022 | 1.01 | 1.00-1.01 |
|  | | | |
| Overall complication | | | |
| Group B | 0.003 | 2.36 | 1.34-4.17 |
| Blood lost | 0.001 | 1.01 | 1.00-1.02 |
|  |  |  |  |
| † a Based on likelihood test adjusted for the other factors in the final model  Abbreviation: BMI, body mass index; ASA-PS, American association of anesthesia-physical status; CCI, Charlson comorbidity index; GB, gallbladder; TG18, Tokyo guideline 18; 95%CI, 95% confidence interval.    Multivariate logistic regression was applied with stepwise backward selection. Initially, all factors presenting with P < 0.5 in the univariate analysis were included in the model. Then factors that showed no or limited statistically significant association (P > 0.1) with overall complication, major complication and non-home discharge adjusted for the remaining factors in the model were deleted from the model in stepwise fashion.  The 9 tested variables for overall complication and major complication were as follows:  Group A or B, Stone impaction of the GB neck, Marked local inflammation, blood lost, operation time,  Subtotal cholecystectomy, TG 18 severity grade Ⅰ or Ⅱ, conversion to open surgery, preoperative GB drainage. | | | |
